# Near-Infrared Spectroscopy Analysis of the Phytic Acid Content in Fuzzy Cottonseed Based on Machine Learning Algorithms

**DOI:** 10.3390/foods13101584

**Published:** 2024-05-20

**Authors:** Hong Yin, Wenlong Mo, Luqiao Li, Yiting Ma, Jinhong Chen, Shuijin Zhu, Tianlun Zhao

**Affiliations:** 1 College of Agriculture and Biotechnology, Zhejiang University, Hangzhou 310058, China; 22116181@zju.edu.cn (H.Y.); 22216136@zju.edu.cn (W.M.); 22016177@zju.edu.cn (L.L.); 22016138@zju.edu.cn (Y.M.); jinhongchen@zju.edu.cn (J.C.); shjzhu@zju.edu.cn (S.Z.); 2 Hainan Institute, Zhejiang University, Sanya 572025, China

**Keywords:** near-infrared spectroscopy, fuzzy cottonseed, phytic acid, models

## Abstract

Cottonseed is rich in oil and protein. However, its antinutritional factor content, of phytic acid (PA), has limited its utilization. Near-infrared (NIR) spectroscopy, combined with chemometrics, is an efficient and eco-friendly analytical technique for crop quality analysis. Despite its potential, there are currently no established NIR models for measuring the PA content in fuzzy cottonseeds. In this research, a total of 456 samples of fuzzy cottonseed were used as the experimental materials. Spectral pre-treatments, including first derivative (1D) and standard normal variable transformation (SNV), were applied, and the linear partial least squares (PLS), nonlinear support vector machine (SVM), and random forest (RF) methods were utilized to develop accurate calibration models for predicting the content of PA in fuzzy cottonseed. The results showed that the spectral pre-treatment significantly improved the prediction performance of the models, with the RF model exhibiting the best prediction performance. The RF model had a coefficient of determination in prediction *(R*^2^*_p_*) of 0.9114, and its residual predictive deviation (RPD) was 3.9828, which indicates its high accuracy in measuring the PA content in fuzzy cottonseed. Additionally, this method avoids the costly and time-consuming delinting and crushing of cottonseeds, making it an economical and environmentally friendly alternative.

## 1. Introduction

Cottonseed is a widely available by-product of cotton processing with high yield potential [1]. Cottonseed contains an array of essential nutrients, such as proteins, oils, fatty acids, and amino acids, making it a valuable raw material for various industrial applications [2]. Through processing, cottonseed can yield valuable products, including cottonseed oil, cottonseed protein, and other derivatives [3]. Cottonseed oil contains essential fatty acids such as linoleic acid and vitamin E. It has good effects in reducing blood cholesterol and antioxidation, making it a high-quality edible oil. It is also one of the main sources of vegetable oil for residents in cotton-producing areas in China. And due to its good stability and crispness, cottonseed oil can be used to make frying oil, shortening, and margarine, and it is used as a substitute for hydrogenated vegetable oil, containing trans fatty acids, in multiple countries. In the industrial sector, cottonseed oil serves as starting material for the synthesis of stearic acid, soft fatty acids, glycerol, and malonic acid, among others [4,5]. Cottonseed is not only a high-quality oil source but also a great protein resource. Many countries have conducted early research on the application of cottonseed protein in food and have developed several edible products. For example, defatted cottonseed protein powder has been added to dried pancakes, donuts, and chocolate candies, and cottonseed protein powder has been widely used as a food additive and flour mixture in cooking [6]. Cottonseed soup is considered a delicacy on the cooking table in some countries on the Asian continent, such as India, Pakistan, and Bangladesh, and the African continent [7]. In addition, the residue of cottonseed after oil extraction, known as cottonseed meal, is used as a fertilizer or a protein source in ruminant animal nutrition. With the research and development of detoxification techniques for cottonseed meal, it has been widely utilized in poultry and monogastric animal feeding, serving as a protein source in feed for chickens, ducks, geese, pigs, and aquaculture [8]. In summary, cottonseed is a by-product with immense potential in various industries, including the food and industrial sectors.

However, cotton itself carries an anti-nutritional factor, phytic acid (PA) [9]. PA is the primary phosphorus (P) storage compound in seeds [10]. The molecular formula of PA is C_6_H_18_O_24_P_6_, and it consists of six phosphate groups and an inositol ring. PA is a valuable raw material used in various industries, including the food, medicine, polymer, metal processing, and fuel industries. However, monogastric animals (such as poultry, swine, and fish) cannot digest phytic acid due to the limited presence or absence of phytase activity in their digestive tract. As a result, these animals release a significant amount of phosphorus into the environment, leading to water pollution through eutrophication [11,12,13]. Furthermore, PA is considered to inhibit the uptake of several essential minerals (especially Fe and Zn), and macro-nutrients like protein, starch, and lipids [14,15].

“Low PA” content as a trait of seeds can provide many potential benefits [13]. Researchers have devoted themselves to reducing their PA content, including using genetic improvements, as well as various pre-treatment methods, such as fermentation, soaking, germination, and the enzymatic treatment of grains with phytase. The rapid and accurate determination of the PA content in a large number of samples is often needed [16]. Currently, the conventional method of high-performance liquid chromatography (HPLC) is the most commonly used technique for measuring PA content [17]. Unfortunately, this method is time-consuming and costly. Furthermore, when preparing cottonseed samples for analysis using this method, the fuzzy cottonseed needs to be delinted with concentrated sulfuric acid, which damages part of the sample, increasing the costs. The discharge of concentrated sulfuric acid waste liquor after depilation also causes the corrosion of pipelines, soil acidification, and other environmental problems.

Rapid and non-destructive near-infrared (NIR) spectroscopy can effectively solve the above problems. NIR spectroscopy is a non-invasive technique used for the analysis of one or more chemical components present in a sample. It entails the utilization of the absorption spectra generated by the combination and overtone bands of select chemical bonds, such as C-H, N-H, O-H, and S-H. This analysis occurs within the near-infrared (NIR) spectral region, ranging between 780 and 2526 nm [18]. However, analyzing NIR spectra can be time-consuming and difficult for researchers to accurately interpret. This is where machine learning comes in. Machine learning algorithms can be trained to map the NIR absorption values to a desired output, such as the concentration of a particular compound in a sample. This process involves both a training phase and a testing phase. During the training phase, the algorithm learns the model parameters by using the recorded NIR spectra as inputs and the desired output as outputs. It can be used during the testing phase to predict the desired output based on new NIR spectra. The accuracy of the model in predicting the desired output for these new spectra can then be evaluated [19]. Overall, machine learning can greatly improve the efficiency and accuracy of NIR spectroscopy analysis, making it a valuable tool.

The combination of NIR spectroscopy and machine learning has broad application prospects in the field of agriculture, the chemical industry, the food industry, and other industries. The combination of NIR spectroscopy and machine learning facilitates rapid, non-destructive inspection of the quality of agricultural products such as crops [20,21,22,23,24,25], fruits [26,27,28], and tea [29,30], allowing for the prediction of various indexes, like moisture, protein, sugar, etc. It can also be applied to classifying, identifying, and evaluating drugs [31]. As a low-cost and rapid method for disease identification, it has also been used for health applications [32]. It can be employed to detect and classify the properties of chemicals [33]. Moreover, it enables the assessment of water contamination and determines the content of organic matter in the soil [34,35]. It is expected that there will be more research on and applications of this technology in the future.

In previous studies, two models for determining the content of phytic acid (PA) in cottonseeds were developed using different calibration methods [36,37]. The studies determined that a least squares support vector machine (LS-SVM) [38] is the most optimal near-infrared calibration model for the prediction of the PA content in cottonseed meal. The LS-SVM model produced a high coefficient of determination in prediction (*R*^2^*_p_*) of 0.97 and a residual predictive deviation (RPD) of 5.53, indicating that the model is highly accurate in predicting the PA content in cottonseed meal. But the samples in these models are subjected to complex processing, which causes damage to them. To address these limitations, a new study was conducted using NIR spectroscopy to measure the PA content in fuzzy cottonseed. This study aimed to develop a more reliable and robust calibration model using NIR spectroscopy combined with different pre-processing and machine learning algorithms, providing an alternative method for detecting the PA content in fuzzy cottonseeds that can replace traditional methods. This study provided insights into the potential of NIR spectroscopy for measuring the PA content in fuzzy cottonseeds.

## 2. Materials and Methods

### 2.1. Samples and Preparation

A total of 456 samples of cottonseeds were gathered from Sanya (18.25° N, 109.30° E), Hainan province, China, in 2020. The cottonseeds we used had a length between 9.5 and 11.0 mm and a width between 4.5 and 6.0 mm, with short fibers left on the surface after cotton shedding.

To ensure the uniformity and stability of the samples, the samples were selected according to the scalding method. After scalding, the samples that were dark brown were gently dried at 37 °C and then moisture-balanced for 2 days.

### 2.2. Near-Infrared Spectroscopy Acquisition

#### 2.2.1. Collection of the NIR Spectra of Fuzzy Cottonseeds

The fuzzy cottonseed samples were scanned for their original spectra using a Büchi NIRFlex-N500 spectrometer (Büchi, Flawil, Switzerland). The samples of fuzzy cottonseed were packed into the solid measuring cell of the apparatus, and the samples were arranged closely in the measuring cell. Each cottonseed sample was placed in the measurement cell three times for scanning, ensuring a similar compactness for each loading to reduce errors. The wavelength range of the near-infrared spectrometer is 1000–2500 nm, and the reflection (R) is collected every 1 nm, with a total of 1501 spectral points. Each sample was measured three times with 64 scans at 25 ± 0.5 °C. After scanning, the spectral data were obtained, the average spectral value of 3 times the spectral data of each sample was calculated, and all the spectra were transformed into absorbance values (log (1/R)).

#### 2.2.2. Collection of the NIR Spectra of the Delinted Cottonseeds

The scanned fuzzy cottonseed samples were delinted using concentrated sulfuric acid, neutralized using NaOH, washed with water, and dried at 37 °C. After water balancing for 2 days, the delinted cottonseed samples were obtained. We repeated the steps in Section 2.2.1 to obtain the NIR spectral data of the delinted seeds.

### 2.3. Determination of the PA Content in the Samples

The weighted least squares support vector machine (WLS-SVM) model established by Zhao [39] was used to determine the content of PA in the samples. The spectral preprocessing method is a combination of three methods in this model: Savitzky–Golay smoothing, standard normal variable transformation, and first derivative transformation. The original spectral data of the delinted cottonseeds were preprocessed using the same method, and then the spectral data were brought into the WLS-SVM model to obtain the PA content (%) of the 456 samples instead of using the traditional determination method.

### 2.4. Construction and Evaluation of the Calibration Model

The Unscrambler v9.7 (CAMOAS, Oslo, Norway) was used to preprocess the spectra, and MATLAB R2021b (MathWorks, Natick, MA, USA) was used to construct and verify the model. Due to the vulnerability of the near-infrared spectrum to non-target factors, such as the sample particle size, baseline drift and offset, light scattering, instrument noise, and ambient environmental factors, various preprocessing techniques were applied to the original spectral data before calibration. These methods included the first derivative (1D) [40], SNV [41], Savitzky–Golay [42] (SG) smoothing, and multivariate scattering correction [43] (MSC) methods. The samples were divided into calibration and prediction sets at a ratio of 3:1 using the Kennard–Stone (KS) algorithm [44]. To build the NIR calibration model for the PA contents in the fuzzy cottonseed samples, the 10-fold cross-validation method was used in combination with linear partial least squares [45] (PLS), support vector machine [46] (SVM), and random forest [47] (RF) modeling methods. The models were evaluated and analyzed according to *R*^2^*_p_*, the RPD, the cross-validation root mean square error (RMSECV), and the root mean square error of prediction (RMSEP). The smaller the RMSECV and the RMSEP, the better the prediction performance and robustness of the model. RPD, defined as the ratio between the standard deviation (SD) of the prediction and the RMSEP, was used to verify the accuracy of the developed calibration models. The higher the value of the RPD, the greater the probability of the model accurately predicting the chemical or physical indices of the sample set. An RPD value greater than 3 can be considered good for predictive purposes. The RPD is commonly used to assess the accuracy of models based on near-infrared spectroscopy.

## 3. Results

### 3.1. Statistical Analysis of the PA Content

Different from the traditional chemical determination methods, the WLS-SVM model uses spectral data from a combination of pre-processing methods to predict the value of the phytic acid content in the samples. Overall, this study highlights the potential for machine learning approaches to be used in analytical chemistry for the accurate and efficient analysis of samples.

**Figure 1:**
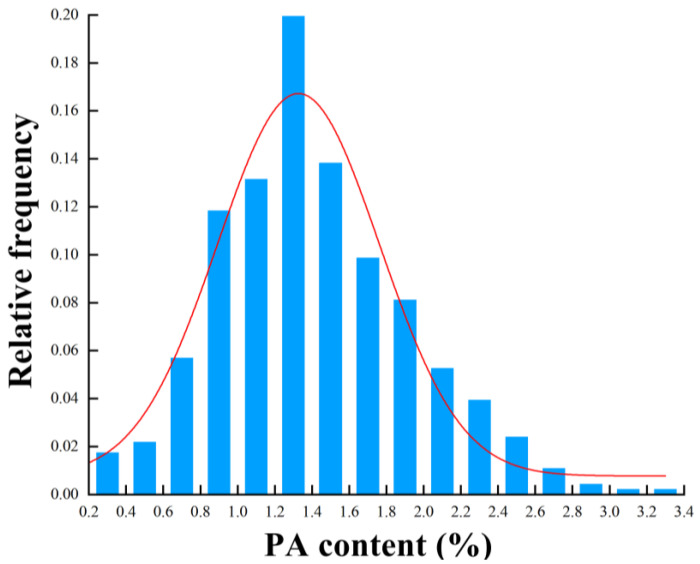
Frequency histograms of PA content for all samples.

The analysis of the PA content in the 456 cottonseed samples is shown in Table 1. Most of the data fell within the range of 0.8% to 1.8%. Fitting with the Gaussian function, the content of PA in the cottonseeds was normally distributed (Figure 1). The average PA content in the samples was found to be 1.92%, with the highest and lowest values at 3.33% and 0.70%, respectively. This indicates a significant 5-fold difference between the maximum and minimum values, with a relatively large standard deviation and a wide content distribution range, providing good representativeness and suggesting significant variation in the PA content among the samples. The Kennard–Stone (KS) algorithm proved to be effective in selecting representative sample subsets, with the calibration set chosen via KS selection providing a better predictive capability compared to the other data selection methods [48]. The KS algorithm was used to divide the samples at a ratio of 3:1, that is, 456 samples were divided into 342 sample correction sets and 114 sample prediction sets. In the NIR models, the calibration set samples covered the PA content range of the prediction set samples, with the mean and standard deviation differences between the two sets being minimal. That is to say, the range of PA content in the calibration set will be wider, and the range of sample PA content in the test set will be fully included in the calibration set, which is sufficient to support the establishment of accurate and robust models.

### 3.2. NIR Spectra and Pre-Treatment

**Figure 2:**
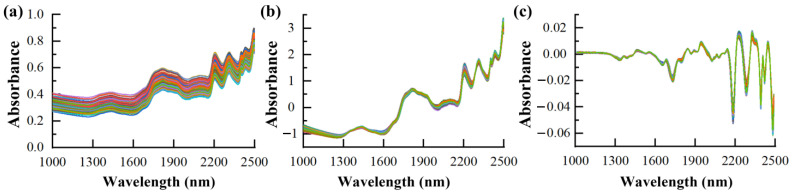
The NIR spectra of the samples. (**a**) The original NIR spectra, (**b**) the NIR spectra processed using SNV, (**c**) the NIR spectra processed using SNV and 1D.

Figure 2a presents the raw spectral curves gathered through the NIR scanning. The chemical bonds of the PA structure for NIR measurement are mainly C-H, P-OH, and O-H bonds [37]. Subsequently, Batten [49] found that the absorption peak attributed to P-OH bonds occurred dominantly at a wavelength of 1908 nm. The spectra showed six dominant absorption peaks around 1613, 1715, 1837, 1964, 2129, and 2356 nm. Notably, the peaks observed at 2129 nm, 1837 nm, and 1964 nm were associated with the combination bands of O-H and P-OH, respectively. Moreover, as NIR detection boasts enhanced spectral stability, hydrogen bonds can substantially facilitate chemical bond analysis of PA in cottonseeds [50].

Throughout the entire spectral range, the absorption peaks and their positions in the spectra of the 456 cottonseed samples remain consistent. However, some notable baseline drift and shift can be observed in the raw spectra. To optimize the processing and construction of calibration models for spectral data, pre-treatment is crucial. Consequently, ten distinct pre-treatment methods were employed to pre-process the fuzzy cottonseed spectra data, and the effects of each method on the raw spectra and PLS models were studied. Model reference indexes for ten representative strategies were evaluated, as shown in Table 2. While not all the pre-treatment methods enhanced the model’s predictive ability, four methods, SG+MSC, SNV+1D, SG+SNV+1D, and SG+MSC+1D, instead diminished the prediction performance of the model. Figure 2b shows the NIR spectra processed using SNV. The parameters show that the pre-treatment methods using a combination of SNV and 1D have the best model parameters, with high *R*^2^*_p_* and RPD values of 0.7865 and 2.1276 and a low RMSEP value of 0.2409. These methods were highly effective in eliminating the effects of baseline, noise, surface scattering, and solid particle size, resulting in significantly enhanced absorption characteristics of the spectra. Figure 2c shows the best combination of pre-treatment results.

### 3.3. Development and Interpretation of Full-Spectrum Models

In this current investigation, a combination of SNV and 1D was applied to construct the linear PLS model, the nonlinear SVM model, and the RF model for predicting the PA content in the cottonseed samples. The results of Table 3 exhibit the parameters of the three models on the PA content in the cottonseed samples. It was observed that the prediction performance of the SVM model and the RF model surpassed that of the PLS model. Among the three models, the RF model was found to be the best NIR-corrected model for predicting the PA content in the cottonseed samples. The RF model produced the highest *R*^2^*_p_* and RPD values of 0.9114 and 3.9828 and the lowest RMSECV and RMSEP values of 0.0747 and 0.1294, respectively.

**Figure 3:**
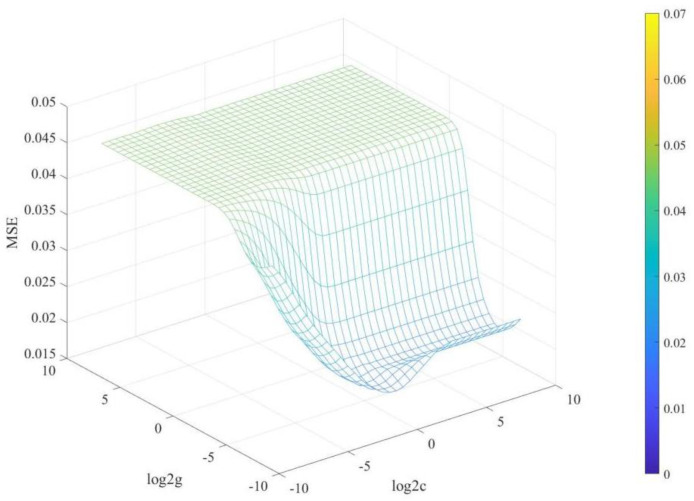
Result of optimal c, g parameter selection.

Furthermore, the study employed the RBF kernel function for SVM modeling, which required fewer parameters, particularly the regularization parameter c and gamma. The complexity of the model was influenced by c and gamma, where higher values of c resulted in more complex models but increased the risk of overfitting. Conversely, lower values of c could lead to underfitting. In addition, the gamma value affected the number of support vectors and the training speed of the model. Specifically, higher gamma values resulted in smaller numbers of support vectors and vice versa. Figure 3 presented the results of the selection of c and gamma parameters using the mean squared error (MSE), where c was found to be 1, and gamma was observed to be 0.0039, giving the best performance.

**Figure 4:**
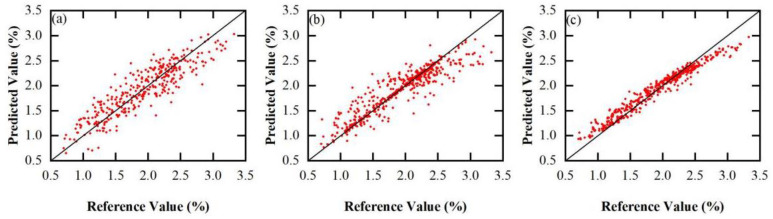
The correlation between predicted and reference values. (**a**) PLS, (**b**) SVM, and (**c**) RF. Samples on diagonal lines indicate their predicted values are equal to reference values.

**Figure 5:**
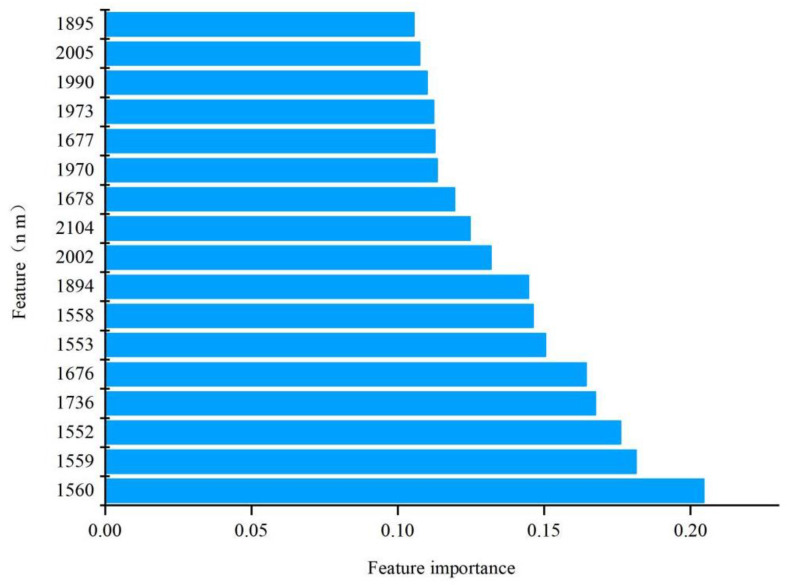
Feature importance ranking using RF.

The regression plots of the predicted and reference values are demonstrated in Figure 4, which showed that the three models represented an acceptable correlation between the predicted and reference values. The diagonal represents the best predicted result, that is, the true value = the predicted value. The closer the sample point is to the diagonal, the better the performance of the model. From Figure 4, it can be seen that the sample distribution of the RF model and the SVM model is closer to the diagonal than the PLS model, indicating that these two models have good predictive performance. The 17 characteristic wavelengths with an importance greater than 0.1 are shown in Figure 5.

## 4. Discussion

Recent research has shown that low-phytate genetics can contribute to mitigating the global eutrophication problem. But this trait can also potentially reduced yields and field performance [15,51]. Developing high-yielding, stress-tolerant crops with a low PA content has become a new challenge for researchers. Because of the low costs and a significant reduction in toxic chemicals, the application of the NIR method could be encouraged and popularized for quantitative determination in agricultural products [48].

In our study, it was feasible to determine the content of PA in the samples using the near-infrared spectroscopy model established by Zhao [37]. In the WLS-SVM model established by Zhao, the phytic acid content of the sample was determined accurately according to HPIC method, and the model established by Zhao was quite accurate. The predictive determination coefficient of the established model reached 0.9768, which means it can completely replace the traditional method of phytic acid content determination. It is feasible to use the established model to determine the phytic acid content of cottonseed instead of the reference method. And other researchers have also used this model to map the QTLs for some traits, which confirms the reliability of this model. Zhao (2020) [52] used this model to determine 13 quality traits, such as the phytic acid content in cottonseed, and to map QTLs for the phytic acid content in cottonseed. The total variation of 22.82–90.44% could be explained by 8 m-QTLs and two pairs of e-QTLs for the phytic acid content.

The delinting process is the separation of the seed from the fiber, and this process does not affect the content of phytic acid in cottonseed because phytic acid is a natural component of the cottonseed, mainly in the endosperm and oil tissue. We established an RF model for fuzzy cottonseed, and the result was also reliable. *R*^2^*_p_* reached 0.9114, also indirectly showing that the model of delinted cottonseed was reliable. 

Due to the large particles in the fuzzy cottonseed samples, there were greater gaps between adjacent samples, which caused a large amount of invalid information in the spectra, thus affecting the prediction accuracy and robustness of the model. Furthermore, since the cottonseed samples were not delinted, their surface was covered with dense fuzz and hard seed shells, which hinder the penetration of NIR light. This, in turn, leads to weakened NIR spectra information and a lower signal-to-noise ratio, thus posing difficulties in processing their feature information. To address these problems, we pre-processed the spectra using 10 strategies to eliminate some invalid information and constructed calibration models. According to the results obtained, it was found that the SNV+1D method had the most favorable pre-treatment effect during the experiment. Selecting the most appropriate pre-processing method presents a challenging task, as almost all of them come with certain drawbacks. The commonly used preprocessing methods in near-infrared spectroscopy include SG, SNV, 1D, MSC, etc. Among them, the SG method is the most commonly used method for eliminating noise, which is used to remove random noise in the near-infrared spectrum and effectively improve the signal-to-noise ratio of the spectrum [42]. SNV transformation is mainly used to eliminate the influence of factors such as light scattering and optical path changes caused by different solid particle sizes and an uneven particle distribution on the spectrum [41]. First-derivative transformation can deduct the influence of the instrument background or drift on the signal and is commonly used to remove baseline offset and superimposed peaks [40]. MSC is commonly used to compensate for additive (baseline shift) and multiplicative effects in the spectral data which are caused by physical effects. For example, non-uniform scattering of the entire spectrum caused by radiation wavelength, particle size, and refractive index [43]. The cotton wool seed samples used in this study have large particles and a dense layer of short fibers on them, which creates certain gaps between the samples and increases the degree of baseline drift. At the same time, the size of the cottonseeds themselves also has a certain impact on the spectrum. These problems can be solved by using SNV and 1D preprocessing. This may be one of the reasons why the most suitable preprocessing method after screening is the combination of SNV+1D [53]. Notwithstanding, there are four techniques that reduced the predictive performance of the model, and there are possible reasons behind it. One of these reasons is the inappropriate adjustment of the pre-treatment parameters. The window size selection in smoothing techniques may affect the size and position of the peaks, thus causing a disturbance to the later quantitative analysis. In this particular experiment, an 11-point window size was used. The second reason for this reduction is excessive pre-treatment. Although the pre-processing step can eliminate noise and impurities, overdosage may lead to the loss of useful information, resulting in a decrease in the model’s effectiveness. The effectiveness of utilizing SG smoothing in pre-processing near-infrared spectra remains a subject of debate, owing to the possibility of losing ambiguous information during this stage [54]. Therefore, it is fundamental to pay attention to the degree of pre-processing to avoid over-pre-treatment. In future experiments, it is necessary to adopt appropriate approaches to selecting and adjusting the pre-treatment techniques and parameters to prevent excessive pre-treatment of the data.

There is inevitably some nonlinear and invalid information in the spectral data of fuzzy cottonseeds. As a powerful approach to eliminating irrelevant variables, pre-treatment could improve the predictive ability and simplify the complexity of the NIR model. The SVM and RF methods are machine learning algorithms that can effectively utilize both linear and nonlinear information for modeling with better robustness. Moreover, the variable importance measure in the RF algorithm can be utilized for the selection of high-dimensional data features. RFs have several advantages, such as their ability to handle both randomized and non-randomized data without overfitting, a fast learning process, and efficient handling of large datasets. Additionally, RFs can manage high-dimensional data without any variable deletion while extracting variable importance information from the data, making them a popular algorithm [55]. 

NIR spectroscopy has demonstrated the capability to effectively quantify the PA content present in fuzzy cottonseeds. However, it is crucial to acknowledge that additional components, particularly in fuzzy cottonseeds, possess C-H, P-OH, and O-H bonds which may have influenced the modeling results. To address this, it is imperative to enhance the accuracy of the NIR model through the application of various machine learning algorithms and other advanced methodologies. At the same time, in actual production, there are also some issues with using NIR technology to measure phytic acid and other components. NIR technology is susceptible to environmental factors such as the sample status and temperature, as well as the influence of operators and instruments. Therefore, if applied in actual production, it is necessary to control environmental factors, and it also requires certain technical requirements for operators. Meanwhile, the models established by different near-infrared machines are not interconnected, which limits the generalization ability of the models. In practical applications, it is necessary to achieve the transfer of calibration models between different near-infrared instruments so that the established calibration models can be applied more widely. At present, although research on NIR technology for the detection of substance content in agricultural products is gradually increasing, the conventional and commercial implementation of this technology is still under development. How to further solve these problems will be important research content in related fields in the future [56,57,58].

## 5. Conclusions

The PA content in fuzzy cottonseed could be rapidly and accurately determined using NIR spectroscopy. The random forest (RF) model, which was designed and optimized based on the spectral pre-processing method of SNV combined with 1D transformation, had the best results. The model showed RMSECV, RMSEP, *R*^2^*_p_*, and RPD values of 0.0747, 0.1294, 0.9114, and 3.9828, respectively, indicating its high accuracy and robustness. Therefore, this approach provides a feasible and effective method for determining the PA content in fuzzy cottonseed. For a long time, people have been committed to reducing the content of phytic acid through breeding and processing. In this process, it is often necessary to quickly and accurately measure the phytic acid content in a large number of samples. The method established in this study also provides a reference for the determination of the phytic acid content in other crops, which will help promote the cultivation of low-phytic-acid crop varieties.

## Figures and Tables

**Table 1:** PA contents in the cottonseeds (%).

Datasets	Min	Max	Average	SD
All samples	0.7029	3.3267	1.9188	0.5473
Calibration	0.7029	3.3267	1.9390	0.5642
Prediction	0.8825	3.1411	1.8582	0.4930

**Table 2:** Parameters of the PLS model established by different pre-treatment methods.

Methods	Model Parameter			
	RMSECV	RMSEP	RPD	*R* ^2^ *_p_*
CK	0.324	0.2785	1.7143	0.6886
SG	0.3203	0.2753	1.7457	0.6968
1D	0.3252	0.256	1.7825	0.7426
SNV	0.3433	0.2456	1.8839	0.746
MSC	0.3316	0.2614	1.7999	0.7017
SG+1D	0.3394	0.2549	1.8261	0.7326
SG+SNV	0.3369	0.2652	1.7952	0.7139
SG+MSC	0.34	0.282	1.7139	0.6794
SNV+1D	0.3637	0.2409	2.1276	0.7865
SG+SNV+1D	0.3198	0.3049	1.4833	0.6353
SG+MSC+1D	0.3304	0.3046	1.4848	0.6309

**Table 3:** Parameters of three models established by PLS, SVM, RF.

Models	Model Parameter			
	RMSECV	RMSEP	RPD	*R* ^2^ *_p_*
PLS	0.3637	0.2409	2.1276	0.7865
SVM	0.1274	0.2207	2.3141	0.8142
RF	0.0747	0.1294	3.9828	0.9114

## Data Availability

The data presented in this study are available on request from the corresponding author due to the raw/processed data required to reproduce these findings cannot be shared at this time, as the data also form part of an ongoing study.
